# Exact Distributions of Finite Random Matrices and Their Applications to Spectrum Sensing

**DOI:** 10.3390/s16081183

**Published:** 2016-07-29

**Authors:** Wensheng Zhang, Cheng-Xiang Wang, Xiaofeng Tao, Piya Patcharamaneepakorn

**Affiliations:** 1 School of Information Science and Engineering, Shandong University, Jinan 250100, China; 2 Institute of Sensors, Signals and Systems, School of Engineering and Physical Sciences, Heriot-Watt University, Edinburgh, EH14 4AS, UK; cheng-xiang.wang@hw.ac.uk (C.-X.W.); pp158@hw.ac.uk (P.P.).; 3 Wireless Technology Innovation Institute, Beijing University of Posts and Telecommunications, Beijing 100876, China; taoxf@bupt.edu.cn

**Keywords:** finite random matrix theory, infinite random matrix theory, eigenvalue distributions, cognitive radio networks, spectrum sensing

## Abstract

The exact and simple distributions of finite random matrix theory (FRMT) are critically important for cognitive radio networks (CRNs). In this paper, we unify some existing distributions of the FRMT with the proposed coefficient matrices (vectors) and represent the distributions with the coefficient-based formulations. A coefficient reuse mechanism is studied, i.e., the same coefficient matrices (vectors) can be exploited to formulate different distributions. For instance, the same coefficient matrices can be used by the largest eigenvalue (LE) and the scaled largest eigenvalue (SLE); the same coefficient vectors can be used by the smallest eigenvalue (SE) and the Demmel condition number (DCN). A new and simple cumulative distribution function (CDF) of the DCN is also deduced. In particular, the dimension boundary between the infinite random matrix theory (IRMT) and the FRMT is initially defined. The dimension boundary provides a theoretical way to divide random matrices into infinite random matrices and finite random matrices. The FRMT-based spectrum sensing (SS) schemes are studied for CRNs. The SLE-based scheme can be considered as an asymptotically-optimal SS scheme when the dimension *K* is larger than two. Moreover, the standard condition number (SCN)-based scheme achieves the same sensing performance as the SLE-based scheme for dual covariance matrix K=2 . The simulation results verify that the coefficient-based distributions can fit the empirical results very well, and the FRMT-based schemes outperform the IRMT-based schemes and the conventional SS schemes.

## 1. Introduction

Cognitive radio networks (CRNs), working as a sharp tool to deal with the spectrum scarcity problem, have been recognized as one of the most promising communication technologies in recent years [1,2,3]. As the key function of CRNs, spectrum sensing (SS) is used to check the accessibility of the target frequency bands, which belong to the primary user (PU) systems, but are not occupied [4,5]. The SS schemes for CRNs have been comprehensively summarized in [2,4,5]. Moreover, the random matrix theory (RMT) has also been used in wireless communication systems [6,7,8,9,10,11].

According to the size of matrix dimensions, the RMT can generally be divided into two types: infinite RMT (IRMT) with large dimensions and finite RMT (FRMT) with finite dimensions. The characteristics of the IRMT have been discussed by some mathematicians, e.g., Alan Edelman [12], Jinho Baik and Jack W. Silverstein [13] and Zhidong Bai [14]. The distributions of the eigenvalues and the condition number (CN) in the IRMT have been expressed as asymptotic formulations, such as the Marchenko–Pastur distributions [15] and the Tracy–Widom distributions [16]. The asymptotic results have been used to construct the SS systems for the CRNs. Based on the eigenvalue distributions of sample covariance matrices, some blind SS schemes have been discussed in [17,18,19]. The eigenvalue ratio in the RMT is a sharp tool to build blind SS schemes.

Compared to the IRMT with large dimensions, the FRMT with finite dimensions has the following advantages in the applications of the practical wireless communication systems. First, the distributions of the FRMT can be evaluated and formulated in the exact and tractable expressions. Second, the FRMT is more suitable for the practical wireless communication systems due to their finite dimensions. However, to the best of our knowledge, the dimension boundary between the IRMT and the FRMT has not yet been precisely defined. We provide the definition of the dimension boundary for Wishart matrices by comparing the theoretical eigenvalue distribution with the empirical eigenvalue distribution.

The FRMT distributions have been discussed in [20,21,22,23,24,25], in which the extreme eigenvalues (largest and smallest) distributions and scaled largest eigenvalue (SLE) distributions were given in [20,21], and [22,23,24], respectively. Our previous work in [25] also provided the extreme eigenvalue distributions based on coefficient matrices. We extend that work and derive the unified distributions of the SLE and the Demmel condition number (DCN) with proposed coefficient matrices (vectors). Note that the FRMT distributions have been given in different expressions, and some of them were not tractable due to high complexity. The unified and compact expressions of the FRMT distributions are required for the applications in the CRNs, because such expressions provide a fast and convenient way to calculate the exact thresholds.

The existing SS schemes can be generally divided into two categories. One is the single SS schemes, where energy detection, feature matched filter detection and cyclostationarity feature detection are usually exploited by the distributed sensors [4,26]; the other is the cooperative SS schemes, where multiple sensors send their sensing samples or sensing results to the fusion centre (FC), and the final sensing result can be determined [27,28]. The cooperative SS schemes can achieve superior sensing performance compared to the single SS schemes. Recently, a robust SS scheme based on crowd sensors, a cooperative SS scheme via Dirichlet process and a multi-agent-based SS framework for 5G networks have been discussed in [29,30,31], respectively. In particular, the random matrix theory (RMT) has been used to construct SS systems that can achieve better sensing performance compared to conventional SS schemes [32,33,34,35,36]. However, the RMT-based SS schemes have to compute the eigenvalues of the sample matrices, leading to extra computational complexity, especially for the IRMT-based schemes.

The FRMT-based SS systems in the CRNs have been discussed in [36], in which the exact SCN distributions of the dual Wishart matrix given in [9] was exploited. The results of [36] indicated that the FRMT-based SS systems can achieve superior sensing performance compared to the counterparts based on the IRMT due to the exact distributions calculated with the FRMT. However, the results of [9] were limited for the dual Wishart matrix, whose dimensions are 2×2 . The exact distributions for the SCN of the Wishart matrix with arbitrary dimensions is still unknown to us, although the sensing performance may be evaluated in an empirical way. Moreover, the sensing performances of other FRMT-based SS systems (e.g., the SLE-based scheme, the largest eigenvalue (LE)-based scheme and the DCN-based scheme) are not clear. This paper aims to address the above problems, and the key contributions are summarized as follows:

1. The exact distributions of the FRMT are unified with the proposed coefficient matrices (vectors). The coefficient reuse mechanism is studied, i.e., the LE distributions and the SLE distributions can be formulated with the same coefficient matrices. Moreover, the SE distributions and the DCN distributions share the same coefficient vectors. In particular, a new and simple CDF of the DCN is formulated with the coefficient vector. The dimension boundary between the IRMT and the FRMT is defined by evaluating the theoretical and empirical eigenvalue distributions.
2. The sensing performances of the FRMT-based SS schemes are analysed and evaluated. The asymptotical optimal SS schemes in the FRMT with varying dimensions are proposed. We demonstrate that the SLE-based scheme is asymptotically optimal when K>2 , and the SCN-based scheme possesses identical sensing performance with the SLE-based scheme when K=2 .

The remainder of the paper is organized as follows: Section 2 presents the system model. The IRMT and FRMT are discussed in Section 3 and Section 4, respectively. The SS systems based on the FRMT are provided in Section 5. The numerical results and analysis are provided in Section 6. This paper is concluded in Section 7.

## 2. System Model

**Figure 1:**
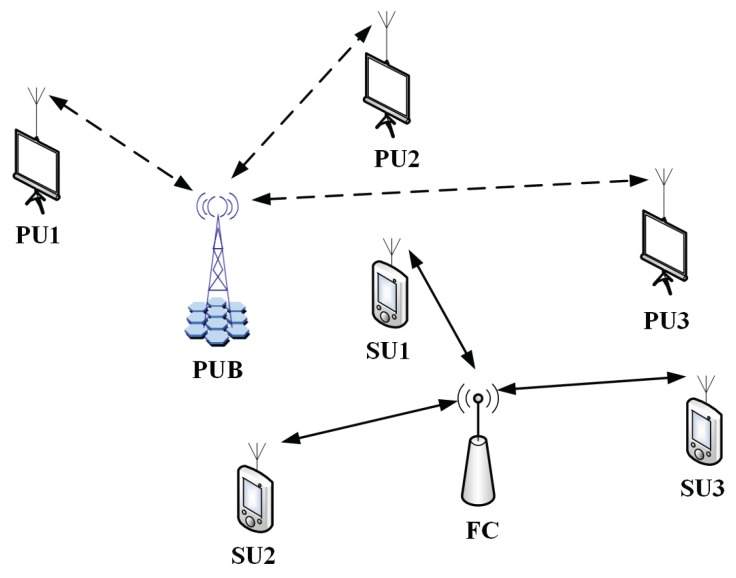
The system model with the PU system consisting of the PU base-station and some PUs (the number is larger than one) and the SU system consisting of the fusion centre (FC) and *K* SUs working as the sensors.

In this section, we introduce the cooperative SS model based on the RMT, followed by some key definitions of the RMT. The system model is shown in Figure 1, in which the secondary user (SU) system working in the interweave CR paradigm [37] dynamically accesses the PU’s spectrum when the PU signals are absent in the target frequency bands. The SUs periodically sense PU signals and send PU samples to the FC. The FC in the SU system periodically gathers the samples from the distributed SUs and makes the final decisions of the availability of the spectrum. Let *K* and *N* denote the number of SUs and the number of the samples of PU signals per SU, respectively. At the FC, a K×K covariance matrix Y can be generated by $Y=X\cdot X^{†}$ , where X denotes the K×N sample matrix and ^†^ denotes transpose-conjugate (Hermitian). Here, we assume that K≤N , and X is full row-rank.

Let $\lambda_{1}\leq \lambda_{2}\leq \cdots \leq \lambda_{K}$ denote *K* ordered eigenvalues of Y . The standard condition number (SCN) denoted by *ξ* is defined as the ratio of the largest eigenvalue $\lambda_{K}$ to the smallest eigenvalue $\lambda_{1}$ , i.e.,

(1) $$\begin{array}{c} \xi ≜\frac{\lambda_{K}}{\lambda_{1}} \end{array}$$

The Demmel condition number (DCN) (the DCN in [38] was defined as the square-root of *κ* given in Equation (2)) denoted by *κ* is defined as the ratio of the trace to the smallest eigenvalue:

(2) $$\kappa ≜\frac{R}{\lambda_{1}}$$

where $R=\text{Tr}\left(Y\right)=\sum_{i=1}^{K}\lambda_{i}$ is the trace of Y The scaled largest eigenvalue (SLE) denoted by *ψ* is defined as the ratio of the largest eigenvalue to the trace, i.e.,

(3) $$\psi ≜\frac{\lambda_{K}}{R}$$

For the IRMT, the critical requirement is that the matrix dimensions should be very large, that is (K,N)→∞ , leading to the asymptotic distributions. Hence, we cannot get the exact distributions of the IRMT due to the large dimensions. The dimension requirements of the IRMT limit its further applications in wireless communication systems based on the following reasons. First, the asymptotic results cannot provide an exact evaluation to the practical systems. Second, the sizes of the practical systems cannot be large enough to satisfy the dimension requirements of the IRMT. Therefore, the FRMT with finite dimensions is more useful for the practical systems because both the number of sensors *K* and the number of the samples per sensor *N* cannot be very large for the practical CRNs. Moreover, the exact and closed-form expressions of the FRMT are required to calculate the exact thresholds in the practical CRNs.

The *i*-th PU signal sample gathered by the *k*-th sensor is:

(4) $$x_{ki}=h_{ki}\cdot s_{ki}+n_{ki},\;\left(k=1,2,\cdots ,K,i=1,2,\cdots ,N\right)$$

where $h_{ki}$ denotes the channel gain between the *k*-th sensor and the FC, the noise $n_{ki}$ denotes an independent and identical distributed (i.i.d.) circular complex Gaussian process with zero mean and variance $\sigma_{n}^{2}$ , the PU signal $s_{ki}$ represents an i.i.d. circular complex Gaussian process with zero mean and variance $\sigma_{s}^{2}$ and the signal-to-noise ratio (SNR) is defined as $\rho =\frac{\sigma_{s}^{2}}{\sigma_{n}^{2}}$ . Therefore, at the FC, a binary hypothesis test (HT) can be formulated as:

(5a) H0:X=Xn(5b) H1:X=Xs+Xn

where $H_{0}$ and $H_{1}$ denote the absence and the presence of the PU signal, respectively, and the *k*-th row of the K×N sample matrix X is the *N*-length sample vector from the *k*-th sensor. The covariance matrix Y can be regarded as a Wishart matrix under the hypothesis $H_{0}$ .

The sensing performance is evaluated by the probability of detection ( $P_{D}$ ) under a given probability of false alarm ( $P_{F}$ ) denoted by *δ*. $P_{D}$ and $P_{F}$ can be formulated as:

(6) $$\begin{array}{c} P_{D}=\text{Pr}\left\{T\geq \gamma |H_{1}\right\} \end{array}$$

(7) $$\begin{array}{c} P_{F}=\text{Pr}\left\{T\geq \gamma |H_{0}\right\} \end{array}$$

where *T* denotes the proposed sensor and *γ* is the given threshold; Pr{·} denotes the probability operation. The threshold *γ* can be calculated with:

(8) $$\begin{array}{c} \gamma ={P_{T}}^{-1}\left(1-\delta \right) \end{array}$$

where $P_{T}^{-1}\left(\cdot \right)$ denotes the inverse function of the cumulative distribution function (CDF) of the sensor *T*. The sensing performance is mainly affected by the threshold *γ*, which is calculated with the distributions of the sensor *T*. This is also the reason why the SS systems based on the FRMT can achieve superior sensing performance compared to those of the IRMT [36]. Based on the RMT, the sensor *T* is constructed by the characteristics of the covariance matrix Y . That is, it is possible to build the sensor with the SCN *ξ*, the DCN *κ*, the SLE *ψ*, and so on. We evaluate the sensors based on various parameters and try to obtain an optimal SS scheme in the FRMT paradigm. For example, if the SCN *ξ* is used to construct the sensor *T*, the formulation is defined as the ratio of the largest eigenvalue $\lambda_{K}$ to the smallest eigenvalue $\lambda_{1}$ :

(9) $$\begin{array}{c} T≜\frac{\lambda_{K}}{\lambda_{1}}=\xi \end{array}$$

For the FRMT, the distributions of the characteristics are critical in the construction of the SS systems because the corresponding thresholds are generated from the CDF of the sensors shown in Equation (8). We unify the distributions with the proposed coefficient matrices (vectors) and provide the simple and compact formulations. The corresponding inverse functions can be generated by the given closed-form expressions.

## 3. Asymptotic Distributions in the IRMT

Before the discussions of the FRMT, we investigate the distributions of the IRMT in this section for comparisons. These results are used to indicate the characteristics of the IRMT when the dimensions are very large, that is K,N→∞ . The dimension boundary between the IRMT and the FRMT is initially defined here to asymptotically divide the IRMT with large dimensions and the FRMT with finite dimensions.

### 3.1. Eigenvalue Distributions in the IRMT

#### 3.1.1. General Eigenvalue Distributions in the IRMT

**Lemma 1 (Marchenko-Pastur Distributions).**  *For a central complex Wishart matrix* W *with the large dimensions (* $K,N\to \infty ,\frac{K}{N}=c$ *), the probability density function (PDF) [15] and the CDF [36] of eigenvalue λ can be formulated as:*

(10) $$\begin{array}{c} f_{\lambda }\left(x\right)=\frac{\sqrt{\left(x-\alpha )(\beta -x\right)}}{2\pi cx} \end{array}$$

(11) $$\begin{array}{c} F_{\lambda }\left(x\right)=\frac{1}{2}+f_{\lambda }\left(x\right)+\frac{\left(1-c\right)}{2\pi }\mathrm{asin}\left(\frac{\left(1+c\right)x-{\left(1-c\right)}^{2}}{2x\sqrt{c}}\right)+\frac{\left(1+c\right)}{2\pi }\mathrm{asin}\left(\frac{1+c-x}{2\sqrt{c}}\right) \end{array}$$

*where the upper bound and lower bound of λ are* $\beta ≜{\left(1+\sqrt{c}\right)}^{2}$ *and* $\alpha ≜{\left(1-\sqrt{c}\right)}^{2}$ *, respectively.*

#### 3.1.2. Extreme (Largest or Smallest) Eigenvalue Distributions in the IRMT

**Lemma 2 (Tracy-Widom Distributions).**  *For a central complex Wishart matrix* W *with the large dimensions (* K,N→∞ , $\frac{K}{N}=c$ *), the PDF and CDF of the centralized and normalized extreme eigenvalue* ${\bar{\lambda }}_{E}$ *can be formulated as:*

(12) $$\begin{array}{c} \;f_{{\bar{\lambda }}_{E}}\left(x\right)=\frac{dF_{\mathrm{TW}2}\left(x\right)}{dx} \end{array}$$

(13) $$\begin{array}{c} \;F_{{\bar{\lambda }}_{E}}\left(x\right)=F_{\mathrm{TW}2}=\exp\left(-\int_{r}^{\infty }\left(x-r\right)q^{2}\left(x\right)dx\right) \end{array}$$

*where* $q^{2}\left(x\right)$ *is the Hastings–McLeod solution of the Painlevé equation of Type II [16,39]. The centralized and normalized extreme eigenvalues are defined as:*

(14) $$\begin{array}{c} {\bar{\lambda }}_{EK}≜\frac{\lambda_{K}-\beta }{\mu } \end{array}$$

(15) $$\begin{array}{c} {\bar{\lambda }}_{E1}≜\frac{\lambda_{1}-\alpha }{\nu } \end{array}$$

*where β and α are defined in Lemma 1; the normalized factors μ and ν are defined as:*

(16) $$\begin{array}{c} \mu ≜\left(\sqrt{K}+\sqrt{N}\right){\left(1/\sqrt{K}-1/\sqrt{N}\right)}^{1/3} \end{array}$$

(17) $$\begin{array}{c} \nu ≜\left(\sqrt{K}-\sqrt{N}\right){\left(1/\sqrt{K}-1/\sqrt{N}\right)}^{1/3} \end{array}$$

### 3.2. Standard Condition Number Distributions in the IRMT

**Lemma 3 (Tracy-Widom-Curtiss Distributions).**  *For a central complex Wishart matrix* W *with the large dimensions (* K,N→∞ , $\frac{K}{N}=c$ *), the PDF [36,40] and the CDF [36] of ξ can be formulated as:*

(18) $$\begin{array}{c} \;f_{\xi }\left(x\right)=\frac{-1}{\mu \;\cdot \;\nu }\int_{0}^{\infty }\;r\;\cdot \;f_{{\bar{\lambda }}_{E}}\;\left(\frac{x\;\cdot \;r-\beta }{\nu }\right)\;\cdot \;f_{{\bar{\lambda }}_{E}}\;\left(\frac{r-\alpha }{\mu }\right)dr \end{array}$$

(19) $$\begin{array}{c} \;F_{\xi }\left(x\right)=\int_{\frac{\alpha }{\mu }}^{\infty }f_{{\bar{\lambda }}_{E}}\left(-r\right)\cdot F_{{\bar{\lambda }}_{E}}\left(\frac{x\cdot (\beta -\mu \cdot r)-\alpha }{\nu }\right)dr \end{array}$$

*where* $f_{{\bar{\lambda }}_{E}}\left(x\right)$ *and* $F_{{\bar{\lambda }}_{E}}\left(x\right)$ *are defined in Lemma 2.*

Seeing the distributions of the IRMT from Lemma 1 to Lemma 3, the expressions are unclosed. This means that only the asymptotic formulations can be exploited to determine the thresholds, leading to low sensing performances.

### 3.3. Dimension Boundary between the IRMT and the FRMT

The dimension boundary is provided to asymptotically divide the IRMT and the FRMT. If the dimensions of a specific random matrix are larger than the dimension boundary, such a matrix can be considered as an infinite random matrix, and the IRMT is therefore applicable. Otherwise, the random matrix with the dimensions less than the dimension boundary is finite, and the FRMT can be used to analyse such a matrix.

**Theorem 1 (Dimension Boundary).**  *For a central complex Wishart matrix* W *with the dimensions K and N, the dimension boundary between the IRMT and the FRMT can be defined as:*

(20) $$\begin{array}{c} \;\left(K_{b},\;N_{b}\right)\;=\;\mathrm{argmin}\;\left\{\;\sum_{\lambda }\left(\left|{ℵ}_{e}\left(K,\;N;\lambda \right)\;-\;{ℵ}_{t}\left(K,\;N;\lambda \right)\right|\right)\right\}\\ \;\text{s.t.}\;0<K\leq N<\infty ,\\ \;K/N=c,\\ \;\lambda \in [\alpha ,\beta ] \end{array}$$

*where* $\left(K_{b},N_{b}\right)$ *denotes the dimension boundary,* ${ℵ}_{e}\left(K,N;\lambda \right)$ *and* ${ℵ}_{t}\left(K,N;\lambda \right)$ *are the empirical and theoretical eigenvalue distributions, respectively,* $c\in \left(0,1\right]$ *is a fixed value and all eigenvalues lie in the eigenvalue interval* [α,β] .

**Proof.**  Under the condition that K,N→∞ and K/N=c , the empirical eigenvalue distributions of the Wishart matrix can be precisely evaluated by the corresponding theoretical eigenvalue distributions. Based on the convergency of the theoretical eigenvalue distribution, the dimension boundary $\left(K_{b},N_{b}\right)$ can always be achieved. When the difference between the two distributions goes to the minimum, the dimension boundary is determined. ☐

Note that Theorem 1 is provided from the view of the IRMT, i.e., when the dimensions of the Wishart matrix are so large that the theoretical eigenvalue distributions of the IRMT can precisely fit the corresponding empirical eigenvalue distributions. Moreover, this theorem can also be defined from the view of the FRMT, and in that case, both the accuracy and the computational complexity of the theoretical eigenvalue distributions in the FRMT should be considered.

However, the dimension boundary determined by Theorem 1 only guarantees that the Wishart matrix with the dimensions larger than such a boundary can be analysed with the IRMT. In practice, there should be a large margin between the IRMT and the FRMT and the Wishart matrices with the dimensions lying in such a margin being able to be handled with both theories. The following corollary provides a scheme to numerically determine the dimension boundary using Theorem 1 and Marchenko–Pastur distributions in Lemma 1.

**Corollary 1 (Dimension Boundary Decision Scheme).**  *For an eigenvalue of the Wishart matrix with the dimensions K and N, the distance between the empirical and theoretical eigenvalue distributions is defined as:*

(21) $$d\left(K,N;\lambda \right)=\left|f_{e}\left(K,N;\lambda \right)-f_{t}\left(K,N;\lambda \right)\right|$$

*where the theoretical eigenvalue PDF* $f_{t}\left(K,N;\lambda \right)$ *can be implemented by the Marchenko-Pastur PDF in Equation (10), and the empirical eigenvalue PDF can be generated in a numerical way. For all of the eigenvalues lying in the eigenvalue interval* [α,β] *, the sum of the eigenvalue distance can be calculated with:*

(22) $$S\left(K,N\right)=\sum_{\lambda \in [\alpha ,\beta ]}d(K,N;\lambda )$$

*The dimension boundary* $\left(K_{b},N_{b}\right)$ *can be achieved if the following condition is satisfied:*

(23) $$\frac{S(K_{b}+\Delta ,N_{b}+\Delta /c)}{S(K_{b},N_{b})}\geq \phi$$

*where Δ denotes the dimension step and ϕ is a given dimension threshold beyond which the corresponding Wishart matrix is considered as infinite.*

**Proof.**  For the theoretical eigenvalue distributions, such as Marchenko-Pastur distributions or Tracy-Widom distributions, when the dimensions get larger, the theoretical eigenvalue distributions converge to the empirical eigenvalue distributions. For the eigenvalues lying in the eigenvalue interval, the sum of the distance between two distributions can be larger than the given dimension threshold for the fixed dimension step. ☐

**Figure 2:**
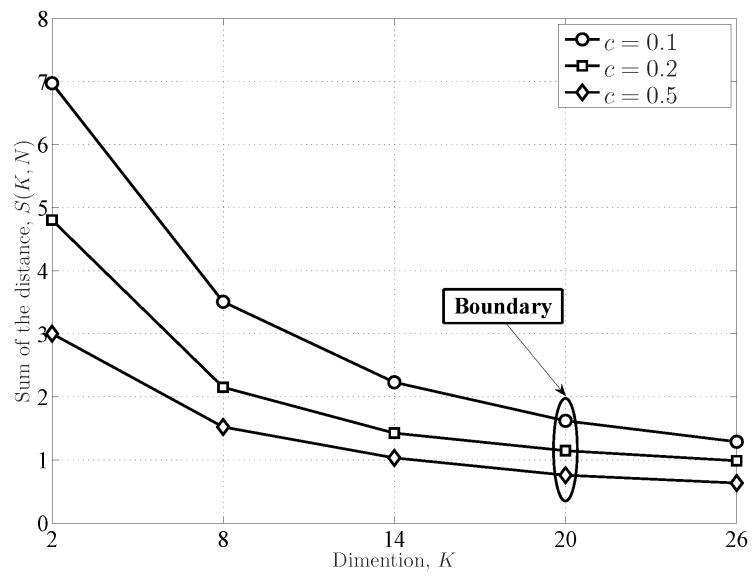
Dimension boundary.

Based on Theorem 1 and Corollary 1, the dimension boundary between the IRMT and the FRMT can be determined. As shown in Figure 2, an example is provided to illustrate the boundary decision process. In the example, the dimensions are set to K=2:6:26 and c=0.1,0.2,0.5 . When the dimension step is Δ=6 and the the dimension threshold is ϕ=0.7 , the dimension boundaries for three cases are (20,200) , (20,100) and (20,40) . Note that for different Δ and *ϕ*, the corresponding boundary should be different.

## 4. Exact Distributions in the FRMT

In this section, we summarize the current research of the FRMT and unify the results with the proposed coefficient matrices (vectors). Based on the coefficient mechanism, the closed-form distributions of the FRMT are derived in exact and compact expressions.

### 4.1. Eigenvalue Distributions in the FRMT

**Lemma 4 (James-Edelman Distribution).**  *The joint PDF of K ordered eigenvalues of* W *is provided by [20,25,41]:*

(24) $$f{\;}_{\Lambda }\;\left(\lambda_{1},...,\lambda_{K}\right)\;=\;\prod_{i=1}^{K}\frac{\lambda_{i}^{N-K}\exp\left(-\lambda_{i}\right)}{(K-i)!(N-i)!}\prod_{1\leq i<j\leq K}\;\;\;\;\;\;{\left(\lambda_{i}-\lambda_{j}\right)}^{2}$$

*where* Λ *denotes the eigenvalue set* $\left\{\lambda_{1},...,\lambda_{K}\right\}$ .

The corresponding joint CDF of *K* ordered eigenvalues of W can be straightforwardly calculated with the definite integral of $f{\;}_{\Lambda }\;\left(\lambda_{1},...,\lambda_{K}\right)$ :

(25) $$F{\;}_{\Lambda }\;\left(\lambda_{1},...,\lambda_{K}\right)\;=\int_{0}^{\lambda_{1}}\cdots \int_{0}^{\lambda_{K}}f_{\Delta }\left(x_{1},\cdots ,x_{K}\right)dx_{1}\cdots dx_{K}$$

However, to the best of our knowledge, there is still no closed-form solution of such a CDF.

### 4.2. Extreme (Largest or Smallest) Eigenvalue Distributions in the FRMT

**Theorem 2 (Largest Eigenvalue Distributions).**  *For a central complex Wishart matrix* W *with finite dimensions, the PDF of the largest eigenvalue* $\lambda_{K}$ *can be formulated in a closed-form formulation [20,25]:*

(26) $$\begin{array}{c} f_{\lambda_{K}}\left(x\right)\;=\;\sum_{k=\;1}^{K}\exp\left(-kx\right)\;\sum_{n=N-K}^{k(N\;+\;K\;-2k)}\;P_{k-1,n-N\;+K}\left(K,N\right)x^{n} \end{array}$$

*where* $P_{k,n}\left(K,N\right)$ *is the* (k,n) *-th entry of the coefficient matrix* P(K,N) .

*The corresponding CDF in a closed-form formulation can be given as [20,25]:*

(27) $$\begin{array}{c} F_{\lambda_{K}}\left(x\right)=\sum_{k=0}^{K}\exp\left(-kx\right)\sum_{n=0}^{k(N+K-2k)}C_{k,n}\left(K,N\right)x^{n} \end{array}$$

*where* $C_{k,n}\left(K,N\right)$ *is the* (k,n) *-th entry of a coefficient matrix* C(K,N) *associated with* P(K,N) .

**Proof.**  Based on the joint PDF of ordered *K* eigenvalues in Equation (24), the CDF of the largest eigenvalue $\lambda_{K}$ can be obtained by integration, that is,

(28) $$F_{\lambda_{K}}\left(x\right)\;=\;\;\int_{0}^{x}\;\;\cdots \;\;\int_{0}^{x}\;\prod_{i=1}^{K}\frac{\lambda_{i}^{N\;-\;K}\;\exp\left(\;-\;\lambda_{i}\right)\det\Omega }{K!(K\;-\;i)!\;(N\;-\;i)!}d\lambda_{1}\;\cdots \;d\lambda_{K}$$

where Ω is the square Vandermonde matrix of $\left\{\lambda_{1},...,\lambda_{K}\right\}$ , and its determinant can be calculated with ([20] Appendix A):

(29)

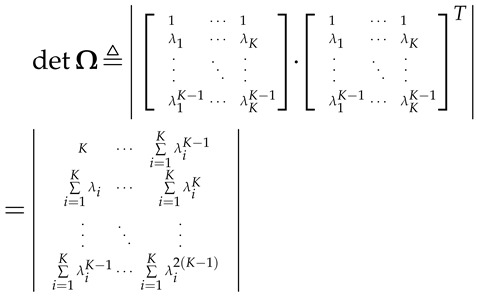

Based on the determinant in Equation (29), the corresponding coefficient matrices P(K,N) for PDF and C(K,N) for CDF can be calculated for the specific *K* and *N*. More details about the generation algorithms for the coefficient matrices can be found in [25], and some typical coefficient matrices and heuristic examples are provided in Appendix A. More coefficient matrices can be calculated with the generation algorithms provided in [25]. ☐

**Theorem 3 (Smallest Eigenvalue Distributions).**  *For a central complex Wishart matrix* W *with finite dimensions, the PDF of the smallest eigenvalue* $\lambda_{1}$ *can be formulated in a closed-form formulation [21,25]:*

(30) $$f_{\lambda_{1}}\left(x\right)=-\exp\left(\;-Kx\right)\sum_{m=N\;-\;K}^{NK-K^{2}}p_{m-(N\;-\;K\;-\;1)}\left(K\;,\;N\right)x^{m}$$

*where* $p_{m-(N\;-\;K\;-\;1)}\left(K\;,\;N\right)$ *is the* $m\;-\;\left(N\;-\;K\;-\;1\right)$ *-th entry of the coefficient vector* p(K,N) .

*The corresponding CDF in a closed-form formulation can be given as [21,25]:*

(31) $$\begin{array}{c} F_{\lambda_{1}}\;\left(x\right)\;=1\;-\;\exp\left(-Kx\right)\;\sum_{m=1}^{NK-K^{2}+1}\;c_{m}\left(K,\;N\right)x^{m\;-\;1} \end{array}$$

*where* $c_{m}\left(K,\;N\right)$ *is the m-th entry of the coefficient vector* c(K,N) *. Different from the coefficient matrices* P[K,N] *and* C[K,N] *, the coefficients* p[K,N] *in Equation* (30) *and* c[K,N] *in Equation* (31) *are coefficient vectors.*

**Proof.**  The proofs for the PDF and CDF can be found in [21,25], and the coefficient vectors are generated in [25]. Some typical coefficient vectors and the corresponding examples are provided in Appendix B. ☐

### 4.3. Condition Number Distributions in the FRMT

#### 4.3.1. Standard Condition Number

**Lemma 5 (SCN Distributions).**  *Let ξ denote the SCN of the central complex Wishart matrix* W *with finite dimensions. The PDF and the CDF of ξ can be formulated as: [9,36]*

(32) $$\begin{array}{c} f_{\xi }\left(x\right)=\Phi \left(N\right)\left[A\left(x;N,N-1\right)+A\left(x;N-2,N+1\right)-2A\left(x;N-1,N\right)\right] \end{array}$$

(33) $$\begin{array}{c} F_{\xi }\left(x\right)=\Phi \left(N\right)\left[B\left(x;N,N-1\right)+B\left(x;N-2,N+1\right)-2B\left(x;N-1,N\right)\right] \end{array}$$

*where:*

(34) $$\Phi \left(N\right)=\frac{1}{2(N-1)!(N-2)!}$$

(35) $$\begin{array}{c} A\left(x;M,N\right)\;=\;\frac{(N\;-\;1)!}{{\left(x\;\;+\;\;1\right)}^{2}}\;\left[\sum_{n=0}^{N\;-\;1}\frac{\left(M\;+\;1\;-\;\frac{n}{x}\right)x^{n}}{{\left(x\;\;+\;\;1\right)}^{n}}\;\;\prod_{m=1}^{M}\;\;\frac{n\;\;+\;\;m}{x\;+\;1}\right]\; \end{array}$$

(36) $$\begin{array}{c} B\left(x;\;M,\;N\right)\;=\;\left(N\;-\;1\right)!\;\left[M!\;\;-\;\;\frac{1}{x\;+\;1}\;\sum_{n\;=\;0}^{N\;-\;1}\frac{x^{n}}{{\left(x\;+\;1\right)}^{n}}\;\;\prod_{m\;=\;1}^{M}\;\;\frac{n\;\;+\;\;m}{x\;\;+\;\;1}\right]\; \end{array}$$

*Note that the above distributions are limited to the dual Wishart matrix* W ( K=2 ).

#### 4.3.2. Demmel Condition Number

**Theorem 4 (DCN Distributions).**  *Let κ denote the DCN of the central complex Wishart matrix* W *with finite dimensions. The PDF of κ can be formulated as [42]:*

(37) $$\begin{array}{c} f_{\kappa }(x)=\Gamma (\frac{m}{2}\sum_{i=N-K}^{(N-K)K}\frac{P_{i-(N-K-1}(K,N)x^{-\frac{m}{2}}}{\Gamma (\frac{m}{2}-i-1){\left(x-K\right)}^{2+i-\frac{m}{2}}} \end{array}$$

*where* m=2KN *and* p(K,N) *is the coefficient vector indicated in Theorem 3;* Γ(n)=n! *denotes the Gamma function.*

*We here provide a new and simple CDF of κ in a closed-form solution using the hypergeometric function instead of the expression in ([42] Equation (18)), which is not tractable due to high complexity:*

(38) Fκ(x)=1-2Γm2x1-m2(m-2)∑i=N-K(N-K)K(-1)i-m2ci-(N-K-1)(K,N)F122-m2+i,1-m2;2-m2;xKΓm2-i-1K2+i-m2

**Proof.**  The CDF of κ is the integral of the PDF in Equation (36), i.e.,

(39) $$F_{\kappa }\left(x\right)≜\int_{0}^{x}f_{\kappa }\left(r\right)dr$$

Considering the key part of Equation (37) $\frac{r^{-\frac{m}{2}}}{{\left(r-K\right)}^{i+2-\frac{m}{2}}}$ , let $-\frac{m}{2}=\mu -1$ , $i+2+\frac{m}{2}=\nu$ and $\beta =-\frac{1}{K}$

Based on the integral of ([43] Equation (3.194.1)):

(40) ∫0xrμ-11+βrνdr=xμμF12ν,μ;1+μ;-βx,

the key part of Equation (38) can be calculated as:

(41) ∫0xr-m2r-Ki+2-m2dr=x1-m21-m2-K2-m2+iF122-m2+i,1-m2;2-m2;xK

Including the corresponding entry of the coefficient vector $c_{i-(N-K-1)}\left(K,N\right)$ and other coefficients of Equation (37), such as $\Gamma \left(\frac{m}{2}\right)$ and $\Gamma \left(\frac{m}{2}-i-1\right)$ , the final CDF of Equation (38) can be calculated after some algebra. Note that the coefficient vector c(K,N) is for the distribution of the smallest eigenvalue $\lambda_{1}$ ; the final CDF of the DCN κ can still use the corresponding coefficients accordingly. ☐

#### 4.3.3. Scaled Largest Eigenvalue

The SLE distributions of the complex and central Wishart matrix W with any dimensions have been discussed in [23,24,44]. The centralized and normalized SLE asymptotically follows the Tracy–Widom distribution in the paradigm that the matrix dimensions should go to infinity [44]. The exact and closed-from distributions of the SLE have been independently given in [23] and [24]. However, the coefficients in the expressions of [24] were calculated with the methods given in [45], which were complicated and not tractable. We unify the results in [23] with the coefficient matrices C[K,N] and P[K,N] given in Theorem 1.

**Theorem 5 (SLE Distributions).**  *Let ψ denote the SLE of the central complex Wishart matrix* W *with finite dimensions. The PDF of ψ can be formulated as [23]:*

(42) $$\begin{array}{c} f_{\psi }\left(x\right)=\frac{\left(\frac{m}{2}-1\right)!}{K^{\frac{m}{2}-1}}\sum_{i=1}^{K}\sum_{j=N-K}^{\left(N+K\right)i-2i^{2}}\frac{{\left(K-ix\right)}^{\frac{m}{2}-j-2}}{(\frac{m}{2}-j-2)!}P_{i,j-(N-K-1)}\left(K,N\right)x^{j}U\left(1-\frac{ix}{K}\right) \end{array}$$

*where* m=2KN , P(K,N) *is the coefficient matrix and* $U\left(x\right)=\left\{\begin{array}{cc} 1, & x\geq 0\\ 0, & x<0 \end{array}\right.$ *denotes the step function. The corresponding CDF is formulated as:*

(43) $$\begin{array}{c} F_{\psi }\left(x\right)=\frac{(\frac{m}{2}-1)!}{K^{\frac{m}{2}-1}}\sum_{i=1}^{K}\sum_{j=N-K}^{\left(N+K\right)i-2i^{2}}i^{\frac{m}{2}-j-2}C_{i,j-(N-K-1)}\left(K,N\right)\Phi \left(x\right) \end{array}$$

*where* C(K,N) *is the coefficient matrix. The function* Φ(x) *is:*

(44) $$\begin{array}{c} \Phi \left(x\right)\;=\;B\left(x\right)U\;\left(\frac{K}{i}\;-\;x\right)\;+\;B\;\left(\frac{K}{i}\right)U\;\left(x\;-\;\frac{K}{i}\right)\;-\;B\left(1\right) \end{array}$$

*and the function* B(x) *is defined as:*

(45) $$\begin{array}{c} \;B\left(x\right)\;=\;{\left(\frac{K}{i}\right)}^{\frac{m}{2}\;-j-\;2}\sum_{q=0}^{\frac{m}{2}\;-j-\;2}\;\frac{{\left(-\frac{i}{K}\right)}^{q}x^{q+j+1}}{\left(\frac{m}{2}\;-\;j\;-\;2\;-\;q\right)!\left(q\right)!\left(j\;+\;q\;+\;1\right)} \end{array}$$

**Proof.**  The proof and the deductions can be found in [23], and the coefficient matrices are shown in Theorem 2. ☐

## 5. SS Schemes Based on the FRMT

In this section, the FRMT-based SS schemes are proposed for the CRNs. In the paradigm of the FRMT, the SLE-based scheme can be considered as an asymptotic generalized likelihood ratio test (GLRT) under the condition that the hypothesis $H_{0}$ is clear and the hypothesis $H_{1}$ is unknown.

### 5.1. SS Schemes Based on Asymptotic GLRT

In a sensing period, the covariance matrix Y can be generated from the sample X , that is $Y=XX^{†}$ . The likelihood ratio (LR) of Y can be written as:

(46) $$\begin{array}{c} L=\frac{f\left(Y;\rho ,\sigma_{n}^{2}|H_{1}\right)}{f(Y;\sigma_{n}^{2}|H_{0})} \end{array}$$

where the channel effect and the PU signal variance $\sigma_{s}^{2}$ can be indicated by SNR ρ. Given a threshold γ, the corresponding GLRT can be expressed as:

(47) $$\begin{array}{c} L_{T}=\frac{{\text{sup}}_{\rho ,\sigma_{n}^{2}}f\left(Y;\rho ,\sigma_{n}^{2}|H_{1}\right)}{{\text{sup}}_{\sigma_{n}^{2}}f\left(Y;\sigma_{n}^{2}|H_{0}\right)}\frac{\overset{H_{1}}{>}}{\underset{H_{0}}{<}}\gamma \end{array}$$

where the symbol sup denotes the supreme. It is reasonable to assume that the noise variance $\sigma_{n}^{2}$ can be estimated in advance for the practical CRNs, and its value is assumed to be unitary, that is $\sigma_{n}^{2}=1$ . In particular, the PU signal SNR ρ is still unknown, and hence, the distributions of Y in the hypothesis $H_{1}$ cannot be obtained. The characteristics of the covariance Y can be indicated by its corresponding eigenvalues.

Loosing the requirements of the GLRT by replacing the distributions of Y with its eigenvalues, we can further revise the GLRT as:

(48) $$\begin{array}{c} L_{T}=\frac{{\text{sup}}_{\rho ,\sigma_{n}^{2}=1}f\left(\lambda_{1},\cdots ,\lambda_{K};\rho |H_{1}\right)}{{\text{sup}}_{\sigma_{n}^{2}=1}f\left(\lambda_{1},\cdots ,\lambda_{K}|H_{0}\right)}\frac{\overset{H_{1}}{>}}{\underset{H_{0}}{<}}\gamma \end{array}$$

where the noise variance is assumed to be unitary. Seeing the right side of the formulation, the eigenvalue PDFs under $H_{0}$ can be obtained from the results given in Section 3 and Section 4. The distributions of the eigenvalues under $H_{1}$ can be asymptotically described by the spiked population model [13,46], in which the large eigenvalues liking the ‘spikes’ can be separated from the bulk of the eigenvalues. However, the number and amplitudes of the ‘spikes’ are hard to estimate. How to construct the ‘right’ sensor that can utilize more information of the covariance matrix Y is the key issue. In other words, how to use the eigenvalues to approximately denote the supremum of the PDF of all eigenvalues under $H_{1}$ , which is a tough problem, needs to be addressed.

The GLRT can be considered as the optimal test under the conditions that the PDFs of Y under $H_{0}$ and $H_{1}$ are clear and all of the parameters such as $\sigma_{n}^{2}$ and ρ are exactly estimated [47]. The conditions are too strict to be satisfied; especially, we cannot obtain the exact distributions of Y under $H_{1}.$ In order to loose the rigid requirements and use the covariance matrix, the characteristics of the covariance matrix Y have been used to construct the SS schemes, which can asymptotically follow the GLRT in the IRMT paradigm [32,33,34,35]. In the IRMT, the largest eigenvalue $\lambda_{K}$ [32], the SCN ξ [33,34] and the SLE κ [35] have been exploited to construct the SS systems, which achieved relatively better sensing performance. Especially, the SLE-based scheme can be considered as the asymptotic GLR statistic ([35] Equation (5)). However, the SLE in the paradigm of IRMT is not suitable for the SS schemes because the SLE distributions in the IRMT cannot be determined.

### 5.2. FRMT-Based SS Schemes

In the FRMT, we have checked the sensing performance of the SCN-based SS scheme and found that the proposed scheme outperforms all of the IRMT-based schemes [36] due to the exact SCN distributions. We use the the exact distributions of the SLE and the SCN to construct the SS systems, and the corresponding sensing performances are evaluated.

The SS algorithm (taking the SCN-based scheme as an example) based on the FRMT can be described as follows:

1. Given the $P_{F}$ ( δ=0.01:0.01:0.99 ), based on the CDF of ξ in Equation (33), the corresponding thresholds are generated by $\gamma ={F_{\xi }}^{-1}\left(1-\delta \right)$ .
2. Construct the sample matrix $X={\left[x_{kn}\right]}_{k=\{1,\cdots ,K\},n=\{1,\cdots ,N\}}$ with K×N PU samples from K sensors, each gathering N samples.
3. Construct the K×K covariance matrix $Y=X\cdot X^{†}$ ; calculate K ordered eigenvalues $\lambda_{1}\leq \lambda_{2}\leq \cdots \leq \lambda_{K}$ ; and let the SCN ξ denote the sensor T, that is $T≜\xi =\frac{\lambda_{K}}{\lambda_{1}}$ .
4. Compare T to the required threshold γ, and record the result $C_{1}=C_{1}+1$ if ξ≥γ ; otherwise, $C_{0}=C_{0}+1$ .
5. Repeat the above operations C times, $C=C_{0}+C_{1}$ , and evaluate the sensing performance by $P_{D}=\frac{C_{1}}{C}$ .

The threshold is the key issue in the construction of the SS scheme based on the RMT. The distributions of the RMT characteristics are required to generate the corresponding threshold. We summarize the distributions of the IRMT and FRMT in Section 3 and Section 4. The results are indicated in the following table. For a given δ, the threshold is generated by $\gamma =F_{T}^{-1}\left(1-\delta \right)$ , where $F_{T}^{-1}\left(x\right)$ is the inverse function of the CDF of T. If the CDF of T is not exact or too complicated, see the CDF of $\lambda_{K}$ in Theorem 3 (Tracy–Widom distributions), we cannot calculate the exact thresholds. Therefore, the sensing performance based on IRMT is not satisfied for the CRNs. However, in the FRMT paradigm, the thresholds can be exactly calculated based on the exact distributions of the sensor *T*; see Table 1. Based on the exact distributions of the FRMT, the corresponding thresholds can be calculated analytically. The eigenvalues are used to constructed the GLRT in Equation (48).

Compared to the SCN-based scheme and the DCN-based scheme, the SLE-based scheme can be considered as an asymptotically optimal SS scheme in the FRMT paradigm based on the following facts:

1. The SLE can be considered as the generalized likelihood ratio (GLR), leading to the fact that the SLE-based scheme is asymptotically GLRT [24,35], which can be considered as an asymptotically optimal SS scheme for the FRMT-based SS schemes;
2. Compared to the SS schemes based on the SCN or the DCN, all eigenvalue information under the hypothesis $H_{1}$ are included;
3. The compact and closed-form distributions of the SLE in the FRMT are available, and the exact thresholds can be generated.

## 6. Numerical Results and Analysis

In this section, the theoretical results, especially the distributions in the IRMT and FRMT, are verified. The sensing performances of the FRMT-based SS schemes are evaluated by the simulations. Note that only the PDFs are indicated to verify the proposed results. The CDFs can be theoretically or numerically generated by the corresponding PDFs, and the verifications of the CDFs are omitted for simplicity.

### 6.1. Theoretical Results’ Verifications

The main distributions in the IRMT and FRMT are verified in this subsection.

#### 6.1.1. IRMT Verifications

The theoretical distributions in the IRMT are verified with the empirical distributions. In order to indicate the characteristics of the IRMT, the dimensions of the random matrices should be very large, that is, K,N→∞ . In Theorem 1 and Corollary 1, the dimension boundary between the IRMT and the FRMT is determined. For Wishart matrices with c=0.1 , when the dimensions are larger than the boundary (20,200) , the theoretical results in the IRMT can work very well. In the following simulations, we set K=30 and N=300 .

**Figure 3:**
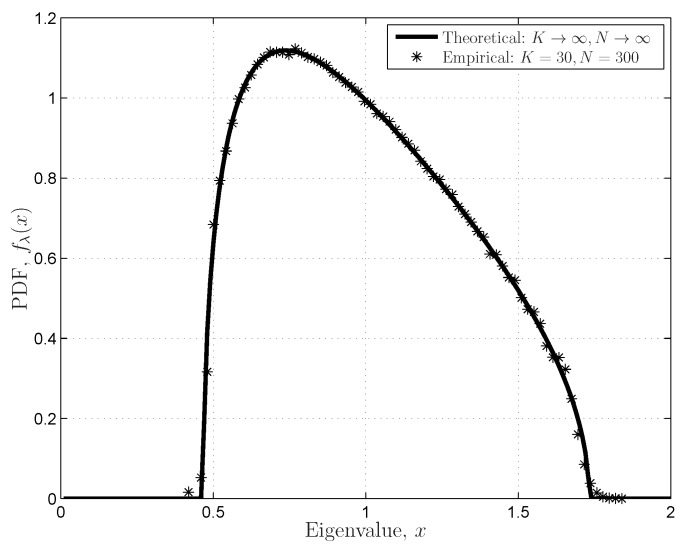
The Marchenko–Pastur distribution (PDF) for the eigenvalues of the central and complex Wishart matrix W with large dimensions.

**Figure 4:**
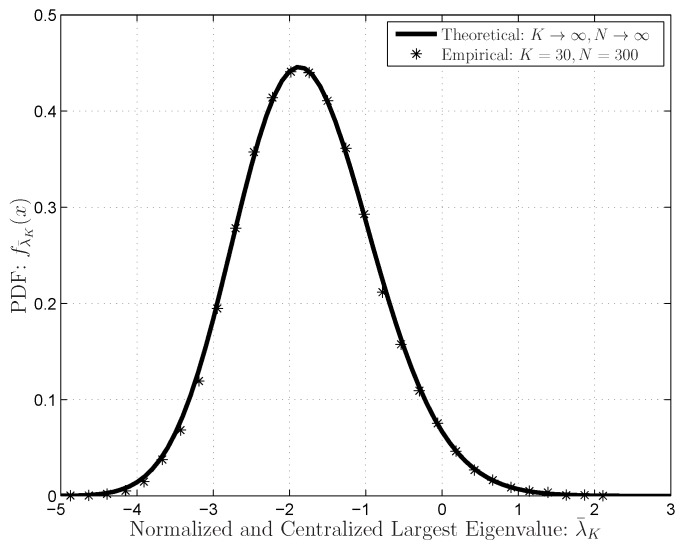
The Tracy–Widom distribution (PDF) for the normalized and centralized extreme (largest) eigenvalue of the central and complex Wishart matrix W with large dimensions.

The asymptotic results of the Marchenko–Pastur distributions and the Tracy–Widom distributions are indicated in Figure 3 and Figure 4, respectively. The results indicate that the theoretical distributions can fit the empirical distributions when the dimensions are very large. Note that the results in ([36], Figure 3) indicated that the Marchenko–Pastur distributions in Equation (10) cannot work well when the dimensions are not very large ( K=5,N=25 ).

#### 6.1.2. FRMT Verifications

**Figure 5:**
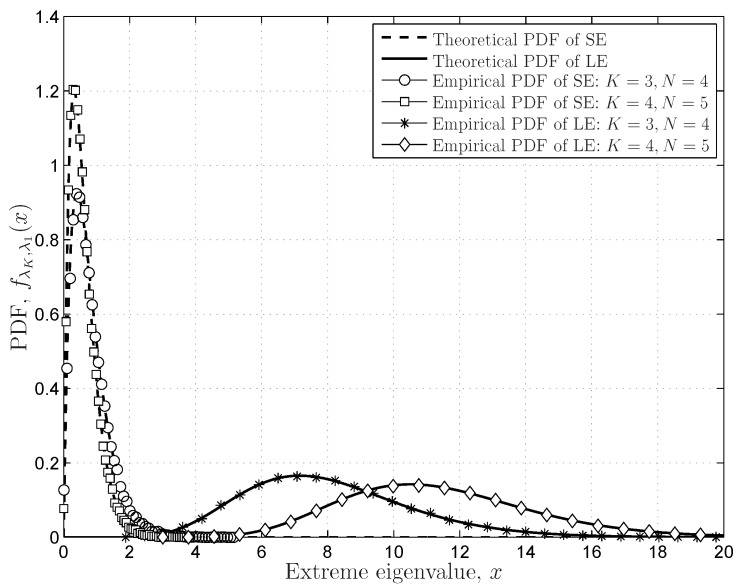
The PDFs of the extreme (largest and smallest) eigenvalues of finite Wishart matrices.

The distributions in the FRMT, including extreme (largest and smallest) eigenvalues, the SCN, the SLE and the DCN, are verified in the following simulations. Figure 5 indicates the distributions of the extreme (largest and smallest) eigenvalues of the central and complex Wishart matrix with finite dimensions ( K=3,4 , N=4,5 ). The theoretical results match the empirical distributions very well, even if the dimensions are finite. The simulations verify the exact distributions in Theorem 2 and Theorem 3 and indicate that the coefficient matrices in the closed-form solutions can work well for the FRMT.

**Figure 6:**
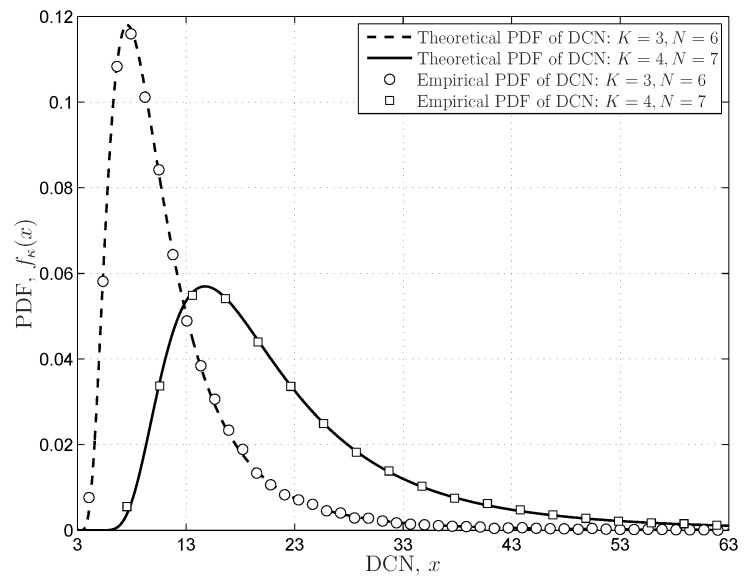
The PDFs of the DCN for the central and complex Wishart matrix W with finite dimensions.

**Figure 7:**
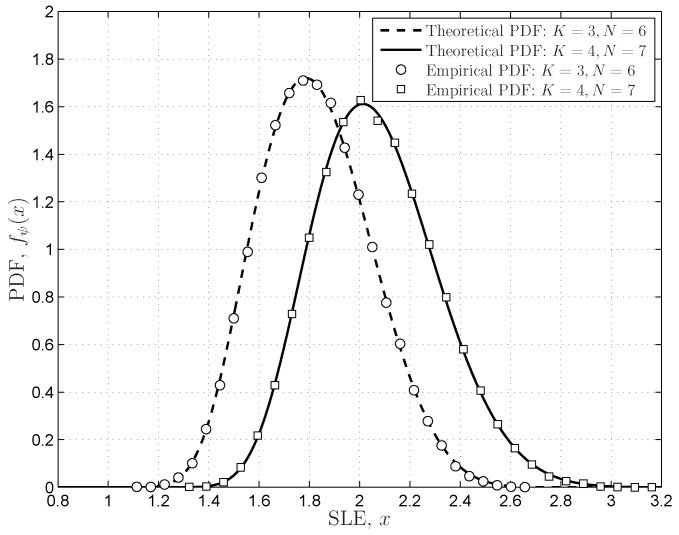
The PDFs of the SLE for the central and complex Wishart matrix W with finite dimensions.

The DCN distributions provided in Theorem 4 are verified in Figure 6, in which the theoretical results match the empirical results very well. The simulations also indicate that the PDF expressions in Equation (37) based on the coefficient vector p(K,N) can precisely describe the DCN PDFs for the finite Wishart matrix. The PDFs of the SLE in Theorem 5 based on the coefficient matrix P(K,N) are verified in Figure 7. We can see that the unified expressions of the SLE match the empirical results very well; even the dimensions are very small ( K=3,N=6 ). The numerical results in Figure 5, Figure 6 and Figure 7 indicate that the coefficient-based expressions with the proposed coefficient matrices (vectors) fit the empirical results very well, even though the matrix dimensions are finite.

### 6.2. Simulations Results for Sensing Performances

**Figure 8:**
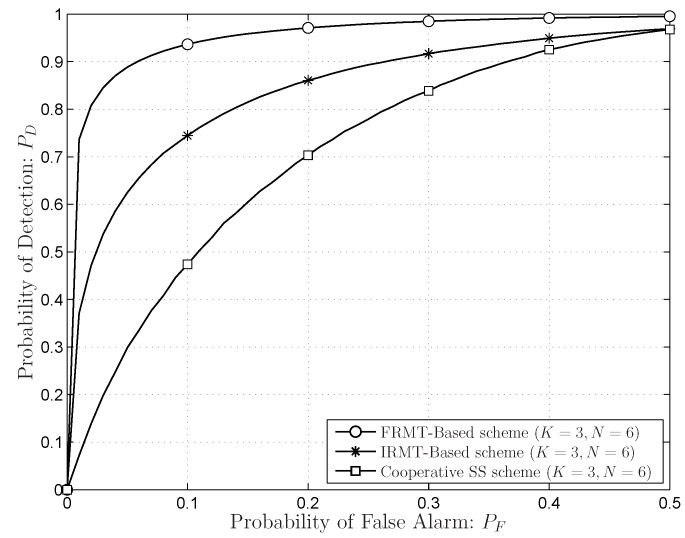
The comparisons of the FRMT-based scheme, the IRMT-based scheme and the conventional cooperative spectrum sensing (SS) scheme. The SNR of PU signal is −5 dB.

The sensing performances of the FRMT-based scheme, the IRMT-based scheme and the conventional cooperative SS scheme are shown in Figure 8, in which the number of the sensors is K=3 , and the sample number per sensor is N=6 . The SNR of PU signal is set to −5 dB. For the FRMT-based scheme, the SLE-based scheme is exploited. The largest eigenvalue calculated with the TW-law of the IRMT is used. For the conventional cooperative SS scheme, the FC gathers PU samples from the distributed sensors and determines the final decision with the energy detection method. The FRMT-based scheme and IRMT-based scheme can achieve better sensing performance compared to the conventional cooperative SS scheme, especially for low $P_{F}$ . Moreover, the FRMT-based scheme is superior to the IRMT-based scheme, because the precise sensing thresholds can be determined by the exact eigenvalue distributions. There is about a 20% performance gain comparing the FRMT-based scheme with the IRMT-based scheme when $P_{F}=0.1$ .

However, the computational complexity of the RMT-based scheme is higher than that of the conventional cooperative SS scheme. The RMT-based schemes have to calculate the eigenvalues of sample matrices. For the SLE-based scheme, all K eigenvalues and the trace R should be calculated. The computational complexity of the eigenvalue calculation is about $O\left({\left(KN\right)}^{3}\right)$ . For the IRMT-based scheme, the largest eigenvalue ${\bar{\lambda }}_{EK}$ of Equation (14) has to be determined. As for the conventional cooperative SS scheme, the computational complexity is about $O\left({\left(KN\right)}^{2}\right)$ under the assumption that the energy detection scheme is used at the FC.

**Figure 9:**
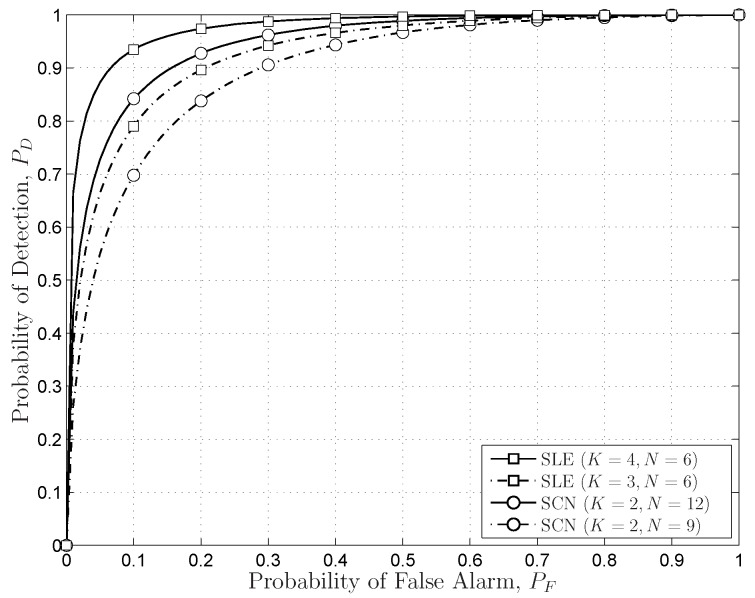
The comparisons of the SLE-based scheme and the SCN-based scheme with the same number of PU samples.

The sensing performances of the proposed SS schemes based on the FRMT are evaluated in the following simulations. The receiver operating characteristic (ROC) performances, the probability of detection ( $P_{D}$ ) against the probability of false alarm ( $P_{F}$ ), are used to evaluate the SS schemes based on the FRMT. Figure 9 indicates that the SLE-based scheme outperforms the SCN-based scheme about 13% when δ=0.1 and the number of PU samples is 24. The thresholds in this simulation are precisely calculated with the exact distributions of the SCN and the SLE.

**Figure 10:**
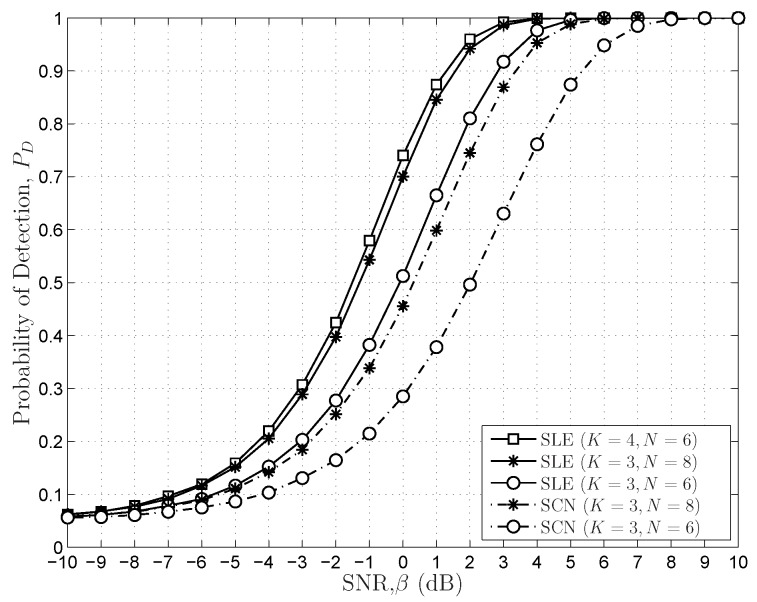
The comparisons of the SLE-based scheme and the SCN-based scheme in the same sensing circumstance, $P_{F}=5\%$ .

Figure 10 compares two SS schemes in the same sensing circumstance when $P_{F}=0.05$ and the total number of the samples is set to 18 and 24. When the SNR is 0 dB and K=3,N=8 , the sensing performances of the SLE-based and SCN-based schemes are about 70% and 45% , respectively. There is about a 25% performance gain. Moreover, the sensing performance of the SLE-based scheme with K=4,N=6 and 0 dB is about 74% , and there is only a 4% performance gain compared to the case of K=3,N=8 . From the results in Figure 10, we can see that the SLE-based scheme outperforms the SCN-based scheme when K>3 under the FRMT paradigm.

**Figure 11:**
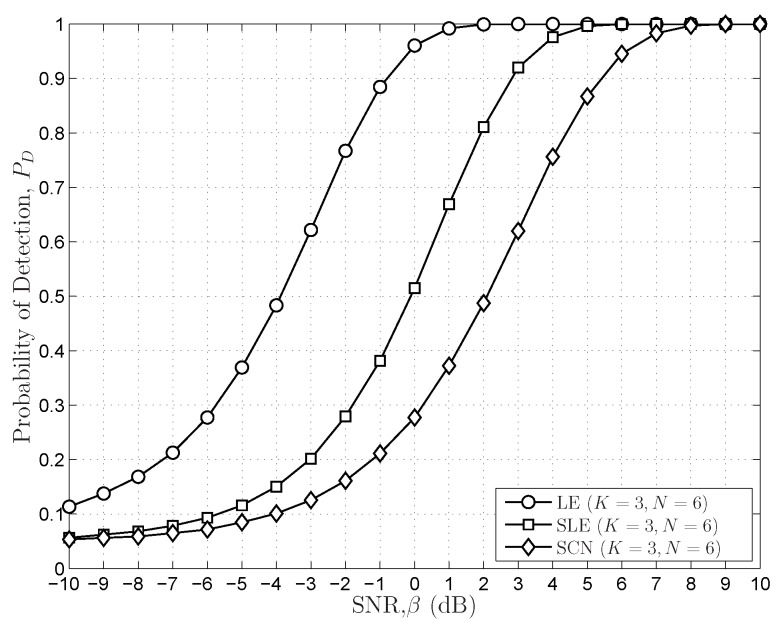
The sensing performances of the LE-based scheme, the SLE-based scheme and the SCN-based scheme for K=3,N=6 . The $P_{F}$ is set to 0.05 .

**Figure 12:**
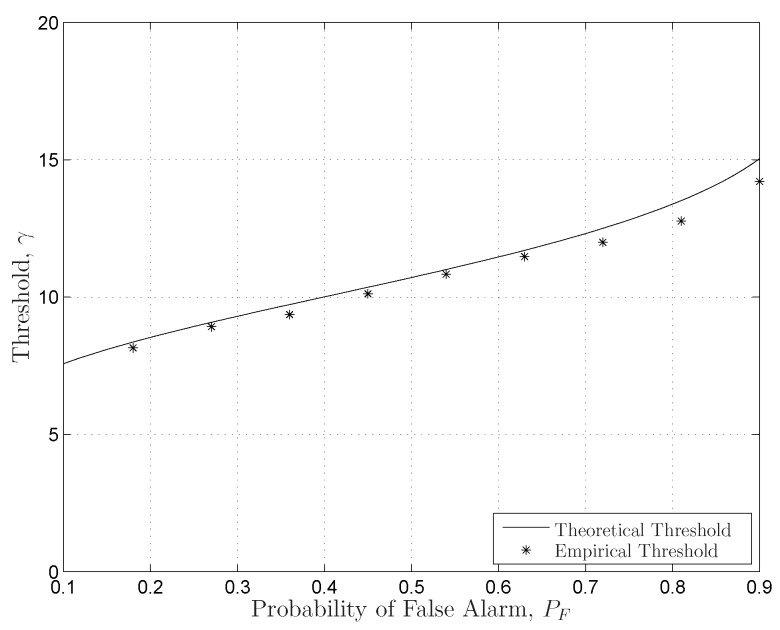
The comparisons of the theoretical thresholds and the empirical thresholds of the LE-based scheme with K=3,N=6 for varying $P_{F}$ . The number of the trials is 10,000.

In Figure 11, the sensing performance of the LE-based scheme is compared to those of the SLE-based scheme and the SCN-based scheme for K=3,N=6 with $P_{F}=0.05$ . The LE-based scheme outperforms both the SLE-based scheme and SCN-based scheme for varying PU SNRs under the condition that the sensing thresholds of the LE-based scheme are predefined in an empirical way. However, like the energy detection scheme, the LE-based scheme also suffers from the noise uncertainty problem, which generates imprecise thresholds. The noise uncertainty problem of the LE-based scheme is illustrated by the simulations in Figure 12, in which the theoretical thresholds generated from the distributions of the LE cannot match the empirical thresholds very well. This means that we cannot calculate the exact thresholds using the distributions of the LE due to the noise uncertainty problem.

## 7. Conclusions

The coefficient-based distributions of the FRMT have been discussed in this paper. The coefficient reuse mechanism has been studied. The same coefficient matrices can be used by the distributions of the SE and the SLE. Moreover, the distributions of the SE and the DCN can share the same coefficient vectors. The FRMT-based SS schemes can achieve better sensing performance, compared to the IRMT-based schemes and the conventional SS schemes. The numerical results have verified that the coefficient-based distributions of the FRMT can fit the empirical results very well. The theoretical analysis and the simulation results have indicated that the SLE-based scheme is an asymptotically optimal scheme in the FRMT paradigm when the dimensions of the Wishart matrix are relatively large, i.e., K>2 .

## Figures and Tables

**Table 1:** Distributions of the random matrix theory (RMT). IRMT, infinite random matrix theory; FRMT, finite random matrix theory; SCN, standard condition number; DCN, Demmel condition number; SLE, scaled largest eigenvalue.

Variable	IRMT		FRMT	
	PDF	CDF	PDF	CDF
Largest Eigenvalue: $\lambda_{K}$	Equation (12)	Equation (13)	Equation (26)	Equation (27)
Smallest Eigenvalue: $\lambda_{1}$	Equation (12)	Equation (13)	Equation (30)	Equation (31)
SCN: *ξ*	Equation (18)	Equation (19)	Equation (32) ^a^	Equation (33) ^a^
DCN: *κ*	PDF of $\sqrt{\kappa }$ ^b^		Equation (37)	Equation (38)
SLE: *ψ*	no results ^c^		Equation (42)	Equation (43)

a, No exact results for K>2 ; b, only for K×K matrices; c, to the best our knowledge, there are no corresponding results in the IRMT.

## Appendix A

The coefficient matrices P(K,N) and C(K,N) for finite dimensions K and N can be calculated with the generation algorithm given in [25]. The coefficient matrices P(K,N) and C(K,N) ( K=3,N≥K ) are indicated in Table A1 and Table A2, respectively. Note that only the cases of K=3,N=3,4,5,6 are provided for simplicity.

**Table A1:** Coefficient matrices P(K=3,N=3,4,5,6).

*N* = 3					*N* = 4					
−3	−6	6	−2	$\frac{1}{4}$	6	−8	$\frac{9}{2}$	−1	$\frac{1}{12}$	0
−6	6	−3	−1	$\frac{-1}{2}$	−12	4	1	−1	$\frac{-1}{12}$	$\frac{-1}{12}$
3	0	0	0	0	6	4	$\frac{1}{2}$	0	0	0
***N* = 5**										
5	−5	2	$\frac{-1}{3}$	$\frac{1}{48}$	0	0				
−10	0	1	0	$\frac{-1}{8}$	0	$\frac{-1}{144}$				
5	5	2	$\frac{1}{3}$	$\frac{1}{48}$	0	0				
***N* = 6**										
$\frac{5}{2}$	−2	$\frac{5}{8}$	$\frac{-1}{2}$	$\frac{1}{240}$	0	0	0			
−5	−1	$\frac{1}{4}$	$\frac{1}{12}$	$\frac{-1}{120}$	$\frac{-1}{120}$	$\frac{1}{2880}$	$\frac{-1}{2880}$			
$\frac{5}{2}$	3	$\frac{13}{8}$	$\frac{1}{2}$	$\frac{7}{80}$	$\frac{1}{120}$	$\frac{1}{2880}$	0			

**Table A2:** Coefficient matrices C(K=3,N=3,4,5,6).

*N*= 3					*N*= 4						
1	0	0	0	0	1	0	0	0	0	0	0
−3	0	−3	1	$\frac{-1}{4}$	−3	−3	$\frac{3}{2}$	$\frac{-13}{6}$	$\frac{7}{12}$	$\frac{-1}{12}$	0
3	0	3	1	$\frac{1}{4}$	3	6	0	$\frac{4}{3}$	$\frac{11}{12}$	$\frac{1}{6}$	$\frac{1}{24}$
−1	0	0	0	0	−1	−3	$\frac{-3}{2}$	$\frac{-1}{6}$	0	0	0
***N* = 5**											
1	0	0	0	0	0	0	0	0			
−3	−3	$\frac{-3}{2}$	$\frac{7}{6}$	$\frac{-23}{24}$	$\frac{5}{24}$	$\frac{-1}{48}$	0	0			
3	6	6	$\frac{2}{3}$	$\frac{1}{3}$	$\frac{1}{3}$	$\frac{1}{9}$	$\frac{1}{72}$	$\frac{1}{288}$			
−1	−3	$\frac{-9}{2}$	$\frac{-17}{6}$	$\frac{-7}{8}$	$\frac{-1}{8}$	$\frac{-1}{144}$	0	0			
***N* = 6**											
1	0	0	0	0	0	0	0	0	0	0	
−3	−3	$\frac{-3}{2}$	$\frac{-1}{2}$	$\frac{1}{2}$	$\frac{-3}{10}$	$\frac{13}{240}$	$\frac{-1}{240}$	0	0	0	
3	6	6	4	$\frac{3}{4}$	$\frac{1}{10}$	$\frac{3}{40}$	$\frac{1}{30}$	$\frac{7}{960}$	$\frac{1}{1440}$	$\frac{1}{5760}$	
−1	−3	$\frac{-9}{2}$	$\frac{-9}{2}$	$\frac{-11}{4}$	$\frac{-21}{20}$	$\frac{-61}{240}$	$\frac{-3}{80}$	$\frac{-1}{320}$	$\frac{-1}{8640}$	0	

Based on the coefficient matrices in the above tables and the corresponding PDF in Equation (26) and CDF in Equation (27), the exact formulations can conveniently be generated. Let K=3 and N=4 , the coefficient matrices P(3,4) and C(3,4) can be given as:

(A1)
$$\begin{array}{c} P\left(3,4\right)=\left[\begin{array}{cccccc} 6 & -8 & \frac{9}{2} & -1 & \frac{1}{12} & 0\\ -12 & 4 & 1 & -1 & \frac{-1}{12} & \frac{-1}{12}\\ 6 & 4 & \frac{1}{2} & 0 & 0 & 0 \end{array}\right] \end{array}$$

(A2)
$$\begin{array}{c} C\left(3,4\right)=\left[\begin{array}{ccccccc} 1 & 0 & 0 & 0 & 0 & 0 & 0\\ -3 & -3 & \frac{3}{2} & \frac{-13}{6} & \frac{7}{12} & \frac{-1}{12} & 0\\ 3 & 6 & 0 & \frac{4}{3} & \frac{11}{12} & \frac{1}{6} & \frac{1}{24}\\ -1 & -3 & \frac{-3}{2} & \frac{-1}{6} & 0 & 0 & 0 \end{array}\right] \end{array}$$

and the corresponding PDF and CDF can be formulated as:

(A3)
$$\begin{array}{c} f_{\lambda_{K}}\left(x\right)=\left(6e^{\;-\;3x}\;-\;12e^{\;-\;2x}\;+\;6e^{\;-\;x}\right)x\;+\;\left(4e^{\;-\;3x}\;+\;4e^{\;-\;2x}\;-\;8e^{\;-\;x}\right)x^{2}\\ +\left(\frac{1}{2}e^{\;-\;3x}\;+\;e^{\;-\;2x}\;+\;\frac{9}{2}e^{\;-\;x}\right)x^{3}\;-\;\left(e^{\;-\;2x}\;+\;e^{\;-\;x}\right)x^{4}\\ -(\frac{1}{12}e^{-2x}-\frac{1}{12}e^{-x})x^{5}-\frac{1}{12}e^{-2x}x^{6} \end{array}$$

(A4)
$$\begin{array}{c} F_{\lambda_{K}}\left(x\right)=1\;-\;e^{\;-\;3x}\;+\;3e^{\;-\;2x}\;-\;3e^{\;-x}\;-\left(\;3e^{\;-\;3x}-6e^{\;-\;2x}\;\;+\;\;3e^{\;-\;x}\right)x\;\\ -\left(\frac{3}{2}e^{\;-\;3x}-\frac{3}{2}e^{\;-\;x}\right)x^{2}\;-\;\left(\frac{1}{6}e^{\;-\;3x}-\frac{4}{3}e^{\;-\;2x}\;+\;\frac{13}{6}e^{\;-\;x}\right)x^{3}\\ +(\frac{11}{12}e^{-2x}+\frac{7}{12}e^{-x})x^{4}+(\frac{1}{6}e^{-2x}-\frac{1}{12}e^{-x})x^{5}+\frac{1}{24}e^{-2x}x^{6} \end{array}$$

## Appendix B

The coefficient vectors p(K,N) and c(K,N) for finite dimensions *K* and *N* are provided in Table B1 as examples. Let K=3 and N=5 , the coefficient vectors p(3,5) and c(3,5) can be given as:

(B1)
$$\begin{array}{c} p\left(3,5\right)=\left[\begin{array}{ccccc} 5 & 5 & 2 & \frac{1}{3} & \frac{1}{48} \end{array}\right] \end{array}$$

(B2)
$$\begin{array}{c} c\left(3,5\right)=\left[\begin{array}{ccccccc} -1 & -3 & \frac{-9}{2} & \frac{-17}{6} & \frac{-7}{6} & \frac{-1}{8} & \frac{-1}{144} \end{array}\right] \end{array}$$

Based on the coefficient vectors and the corresponding PDF in Equation (30) and CDF in Equation (31), the corresponding formulations can conveniently be written as:

(B3)
$$\begin{array}{c} \;f_{\lambda_{1}}\left(x\right)\;=\;5e^{\;-3x}x^{2}\;+\;5e^{\;-\;3x}x^{3}\;+\;2e^{\;-3x}x^{4}\;+\;\frac{1}{3}e^{\;-3x}x^{5}\;+\;\frac{1}{48}e^{\;-3x}x^{6} \end{array}$$

(B4)
$$\begin{array}{cc} F_{\lambda_{1}}\left(x\right) & =1\;-e^{\;-3x}\;-3e^{\;-3x}x\;-\frac{9}{2}e^{\;-3x}x^{2}\;-\frac{17}{6}e^{\;-3x}x^{3}\\ & -\frac{7}{6}e^{\;-3x}x^{4}\;-\frac{1}{8}e^{\;-3x}x^{5}\;-\frac{1}{144}e^{\;-3x}x^{6} \end{array}$$

**Table B1:** Coefficient matrices p(K=3,N=3,4,5,6) and c(K=3,N=3,4,5,6) .

Coefficient Matrices p (K=3,N=3,4,5,6)									
***N* = 3**	***N* = 4**			***N* = 5**					
3	6	4	$\frac{1}{2}$	5	5	2	$\frac{1}{3}$	$\frac{1}{48}$	
***N* = 6**									
$\frac{5}{2}$	3	$\frac{13}{8}$	$\frac{1}{2}$	$\frac{7}{80}$	$\frac{1}{120}$	$\frac{1}{2880}$			
**Coefficient Matrices c** (K=3,N=3,4,5,6)									
***N* = 3**	***N* = 4**								
−1	−1	−3	$\frac{-3}{2}$	$\frac{-1}{6}$					
***N* = 5**									
−1	−3	$\frac{-9}{2}$	$\frac{-17}{6}$	$\frac{-7}{6}$	$\frac{-1}{8}$	$\frac{-1}{144}$			
***N* = 6**									
−1	−3	$\frac{-9}{2}$	$\frac{-9}{2}$	$\frac{-11}{4}$	$\frac{-21}{20}$	$\frac{-61}{240}$	$\frac{-3}{80}$	$\frac{-1}{320}$	$\frac{-1}{8640}$
